# A Case of Severe Acute Gallstone Pancreatitis With Black Ascites in a Patient Without Underlying Diseases

**DOI:** 10.7759/cureus.82807

**Published:** 2025-04-22

**Authors:** Toshimitsu Kobori, Ginga Suzuki, Yoshimi Nakamichi, Hibiki Serizawa, Saki Yamamoto

**Affiliations:** 1 Critical Care Center, Toho University Omori Medical Center, Tokyo, JPN

**Keywords:** acute gallstone pancreatitis, black ascites, black pancreatic ascites, ercp, pancreatic ascites

## Abstract

In acute pancreatitis, ascitic fluid is typically pale yellow and exudative due to inflammation. We report a rare case of black ascitic fluid associated with gallstone-induced severe acute pancreatitis in a 71-year-old man with no underlying disease. The patient initially presented to a local hospital with acute-onset abdominal pain. Abdominal computed tomography (CT) revealed a common bile duct stone, and he was diagnosed with acute cholangitis. Endoscopic retrograde cholangiopancreatography (ERCP) with stone extraction and endoscopic nasobiliary drainage (ENBD) was performed. On the following day, his abdominal pain worsened, and further evaluation revealed elevated pancreatic enzyme levels and peripancreatic inflammatory changes on CT. He was subsequently diagnosed with acute pancreatitis. Despite four days of treatment with fluid resuscitation and antibiotics, his condition deteriorated, and he was transferred to our facility on Day 0. Upon arrival, he was intubated and started on mechanical ventilation due to respiratory failure. Continuous hemodiafiltration (CHDF) was initiated on Day 1 for metabolic acidosis and worsening renal function. On Day 3, abdominal CT revealed newly developed ascites, and paracentesis was performed due to concern for increased intra-abdominal pressure. The ascitic fluid appeared black. Laboratory analysis revealed a mildly elevated total bilirubin level and markedly elevated amylase and lipase levels, consistent with pancreatic ascites. Despite drainage and intensive supportive care, the patient developed multiple organ dysfunction syndrome (MODS), including refractory shock, respiratory failure, and renal insufficiency. He died on Day 5 of hospitalization. This case highlights an extremely rare presentation of pancreatic ascites with black discoloration in the early phase of acute pancreatitis. The black color was most likely due to pancreatic duct disruption and enzyme-mediated hemorrhagic changes. To our knowledge, this is the first reported case of black pancreatic ascites secondary to acute pancreatitis. Awareness of this rare manifestation may aid in the timely recognition and management of similar cases in the future.

## Introduction

Acute pancreatitis is an inflammatory condition caused by the autodigestion of pancreatic and peripancreatic tissues by pancreatic enzymes. Common etiologies include gallstones, alcohol use, hyperlipidemia, medications, postoperative complications, and infections. Among these, gallstone-induced pancreatitis is one of the most frequent causes, accounting for approximately 35%-40% of all acute pancreatitis cases [1,2].

The differential diagnosis of gallstone pancreatitis includes other causes of acute abdomen, such as acute cholecystitis, cholangitis, peptic ulcer disease, biliary colic, and acute myocardial infarction. Appropriate diagnostic tools include abdominal ultrasonography, computed tomography (CT), magnetic resonance cholangiopancreatography (MRCP), and liver function tests.

Initial management of gallstone pancreatitis includes aggressive fluid resuscitation, analgesia, nutritional support, and antibiotic administration when indicated. In cases with concomitant cholangitis or suspected biliary obstruction, early endoscopic retrograde cholangiopancreatography (ERCP) is recommended [2]. Severe cases may progress to complications such as multiple organ dysfunction syndrome (MODS), pancreatic necrosis, infected pancreatic collections, systemic inflammatory response syndrome (SIRS), and ascitic fluid accumulation.

Although ascites is not uncommon in the context of acute pancreatitis, it typically presents as pale yellow exudative fluid due to inflammation. In contrast, ascitic fluid resulting from leakage of pancreatic secretions is referred to as pancreatic ascites, which may occasionally appear black. Pancreatic ascites is usually associated with chronic pancreatitis, particularly of alcoholic origin, and is characterized by markedly elevated levels of pancreatic amylase in the fluid [3].

The macroscopic appearance of ascitic fluid can provide useful diagnostic clues. Among the rarest findings is black ascites, which has been reported in association with conditions such as bowel perforation with fecal contamination, fungal peritonitis, metastatic melanoma, primary ovarian cancer, and pancreatic ascites [4-9]. When black ascites is encountered, it is essential to thoroughly evaluate the underlying pathology.

However, to the best of our knowledge, there have been no prior reports of black pancreatic ascites secondary to acute pancreatitis. Herein, we report a rare case of severe gallstone-induced acute pancreatitis in a patient without underlying disease, presenting with black pancreatic ascites.

## Case presentation

**Figure 1:**
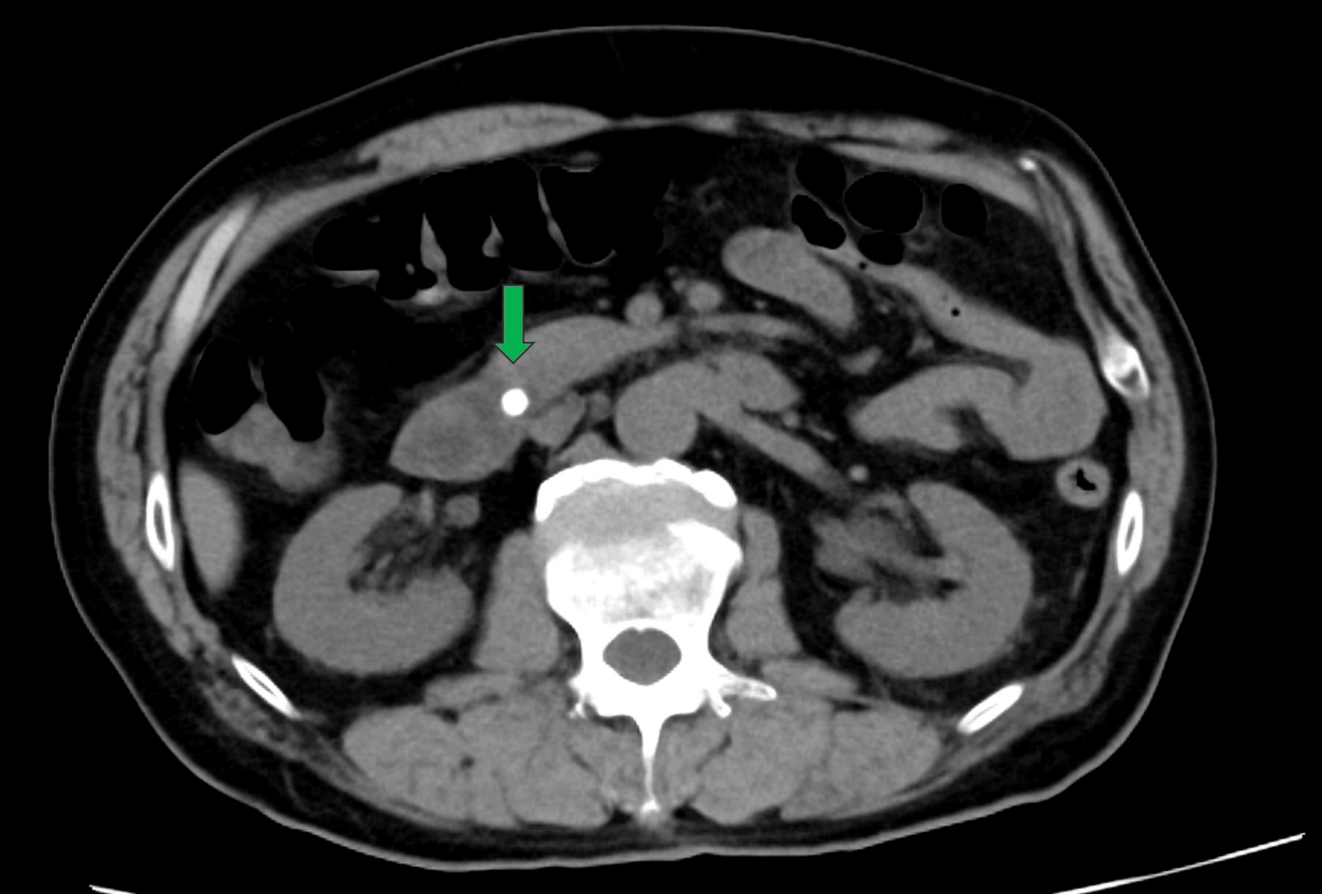
Abdominal CT performed at the referring hospital demonstrating a common bile duct stone (green arrow) without evidence of ascites CT: computed tomography

A 71-year-old man with no prior medical history was transferred to our hospital for the management of severe acute pancreatitis caused by gallstones. He had no history of alcohol use, viral infection, or recent vaccination. Abdominal computed tomography (CT) performed at the referring hospital revealed a common bile duct stone (Figure 1), and laboratory tests showed elevated C-reactive protein and pancreatic amylase levels. The patient was diagnosed with acute cholangitis and severe acute gallstone pancreatitis. Endoscopic retrograde cholangiopancreatography (ERCP) with stone removal and endoscopic nasobiliary drainage (ENBD) was performed.

**Figure 2:**
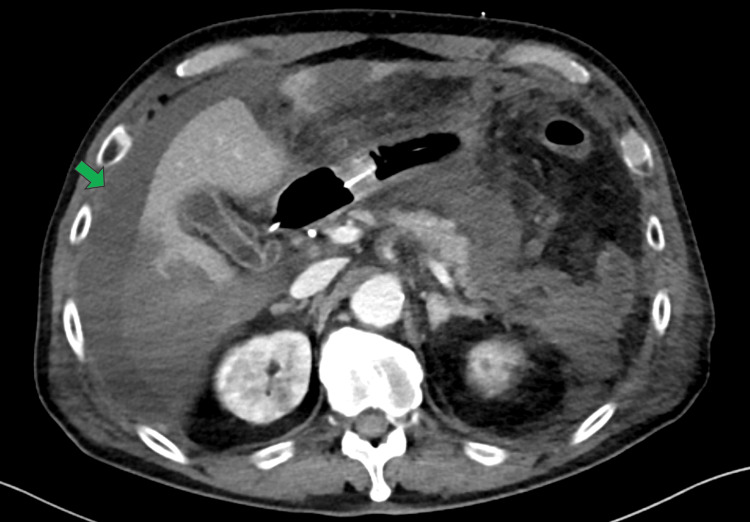
Abdominal CT on admission to our hospital showing new-onset ascites (green arrow) without evidence of a pancreatic pseudocyst CT: computed tomography

Despite four days of treatment at the referring hospital, including antibiotics and fluid resuscitation, his condition worsened, and he was transferred to our facility. Upon admission (Day 0), he was intubated due to respiratory failure and started on mechanical ventilation. Laboratory findings at admission are shown in Table 1. Abdominal CT at our hospital revealed mild ascites without evidence of a pancreatic pseudocyst (Figure 2). Continuous hemodiafiltration (CHDF) was initiated on Day 1 for worsening renal function and metabolic acidosis.

**Table 1:** Laboratory findings on admission to our hospital *Abnormal WBC: white blood cell count, CRP: C-reactive protein, Hb: hemoglobin, Plt: platelets, AST: aspartate aminotransferase, ALT: alanine aminotransferase, ALP: alkaline phosphatase, γ-GTP: gamma-glutamyl transpeptidase, T-Bil: total bilirubin, Alb: albumin, BUN: blood urea nitrogen, Cre: creatinine, Na: sodium, K: potassium, Cl: chloride, PT: prothrombin time, APTT: activated partial thromboplastin time

Laboratory examination	Result	Reference range
WBC	14,100/μL*	3,500-9,000/μL
CRP	28 mg/dL*	0.0-0.3 mg/dL
Hb	11.7 g/dL*	Male: 13.5-17.5 g/dL/female: 12-16 g/dL
Plt	45,000/μL*	150,000-350,000/μL
AST	5,674 U/L*	10-40 U/L
ALT	2,651 U/L*	5-45 U/L
ALP	183 U/L	100-350 U/L
γ-GTP	78 U/L*	Male: 10-70 U/L/female: 7-32 U/L
T-Bil	6.5 mg/dL*	0.2-1.2 mg/dL
Alb	1.9 g/dL*	3.8-5.3 g/dL
BUN	53 mg/dL*	8-20 mg/dL
Cre	2.89 mg/dL*	Male: 0.7-1.1 mg/dL/female: 0.5-0.8 mg/dL
Na	131 mEq/L*	135-145 mEq/L
K	4.3 mEq/L	3.5-5.0 mEq/L
Cl	103 mEq/L	98-108 mEq/L
Calcium (corrected)	3.9 mg/dL*	8.5-10.5 mg/dL
Serum amylase	3,429 U/L*	40-120 U/L
Glucose	149 mg/dL*	70-109 mg/dL
PT	13 seconds	10-13 seconds
APTT	39.3 seconds*	25-35 seconds

**Figure 3:**
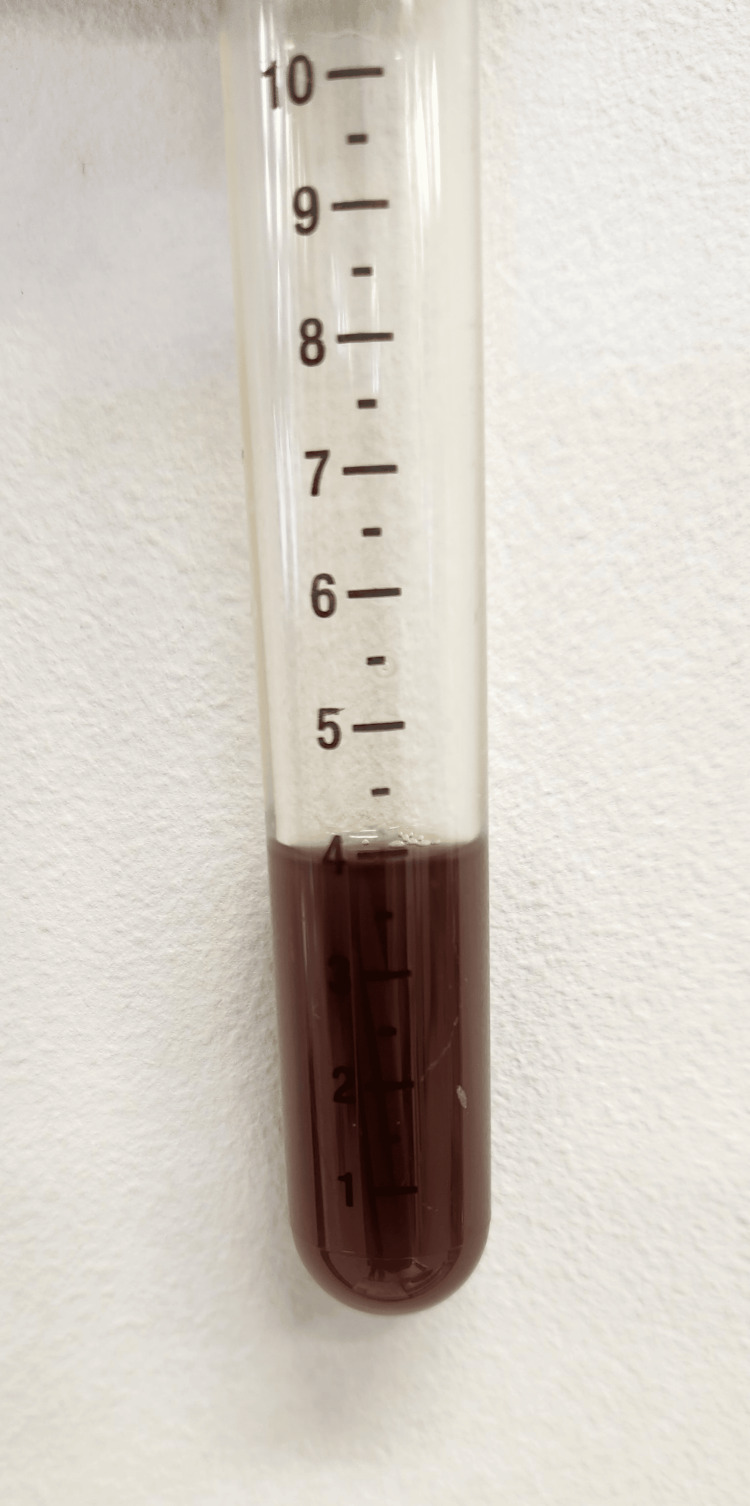
Black ascitic fluid

On Day 3, paracentesis was performed to reduce intra-abdominal pressure. The aspirated ascitic fluid appeared black (Figure 3). Fluid analysis revealed a mildly elevated total bilirubin level and a markedly elevated amylase level, consistent with pancreatic ascites (Table 2). Although drainage was effective, the patient's condition continued to deteriorate.

**Table 2:** Ascitic fluid analysis on Day 3 showing markedly elevated amylase levels, consistent with pancreatic ascites P-AMY: pancreatic amylase, T-Bil: total bilirubin

Parameter	Result	Reference range/interpretation
Appearance	Turbid	-
Color	Black	-
Amylase	3,608 U/L	<100 U/L
P-AMY	2,819 U/L	<100 U/L (serum-like) (>1,000 U/L suggests pancreatic origin)
Lipase	3,501 U/L	<60 U/L
T-Bil	1.7 mg/dL	<1.2 mg/dL
Culture	Negative	Negative (positive suggests infection)

By Day 4, he had developed multiple organ dysfunction syndrome (MODS), which is defined as the progressive dysfunction of two or more organ systems requiring medical intervention to maintain homeostasis. In this case, MODS was characterized by worsening respiratory failure requiring continued mechanical ventilation, refractory hypotension despite vasopressor use, and progressive renal dysfunction necessitating continuous hemodiafiltration (CHDF). Despite appropriate fluid resuscitation, ventilatory support, and renal replacement therapy, his oxygen demand and vasopressor requirements continued to rise, indicating persistent deterioration of both circulatory and respiratory function. Despite intensive supportive care, he died on Day 5 due to progressive MODS.

**Figure 4:**
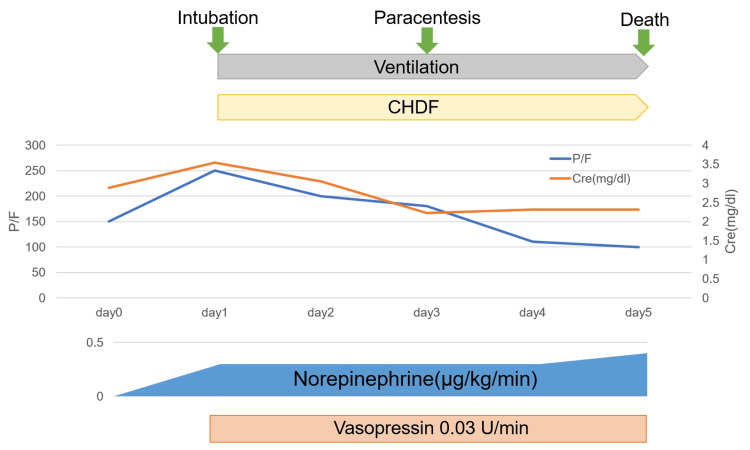
Clinical timeline CHDF: continuous hemodiafiltration

A summary of the clinical timeline is shown in Figure 4.

## Discussion

The notable feature of this case is the presence of black pancreatic ascites in a patient with severe gallstone-induced acute pancreatitis without underlying disease. To the best of our knowledge, this is the first reported case of its kind.

Pancreatic ascites is typically associated with chronic pancreatitis, particularly of alcoholic origin [3,7]. In acute pancreatitis, inflammation may lead to pancreatic duct leakage, which is usually contained by adjacent anatomical structures such as the posterior gastric wall, transverse colon, and mesocolon, resulting in the formation of pancreatic pseudocysts. When a pseudocyst ruptures into the peritoneal or pleural cavity, ascites or pleural effusion may develop. The severity of these complications often correlates with the size of the pseudocyst [1].

In the present case, ascitic fluid analysis revealed a mildly elevated total bilirubin level and a markedly elevated pancreatic amylase level, consistent with pancreatic ascites. Given the early phase of the disease, it was unlikely that a pseudocyst had formed and ruptured. Instead, pancreatic duct disruption with direct leakage of pancreatic secretions into the peritoneal cavity was considered the most plausible mechanism.

The black discoloration of the ascitic fluid, while extremely rare, warrants special consideration. Several mechanisms have been proposed in the literature, including intraperitoneal bleeding due to enzymatic vascular injury, the accumulation of necrotic tissue, and the presence of hemoglobin degradation products such as methemalbumin [4-7]. Additional case reports have described black ascitic fluid in association with chronic pancreatitis or malignancy, although the exact mechanisms are often unclear [7,8]. In very rare cases, fungal infections such as *Aspergillus* peritonitis or malignant melanoma have also been reported as potential causes [9]. In our case, cultures were negative, and there were no clinical or laboratory findings clearly suggestive of infection or malignancy. Although malignancy could not be entirely ruled out, the markedly elevated ascitic amylase level and the absence of other explanatory factors led us to conclude that the black discoloration was most likely due to enzyme-mediated hemorrhagic changes secondary to pancreatic duct disruption in acute pancreatitis.

Compared with typical ascitic fluid, which is clear or straw-colored, pancreatic ascites is often turbid and has amylase levels significantly above the normal range. In our patient, the amylase level in the ascitic fluid exceeded 3,608 U/L, far above the typical upper limit of approximately 100 U/L in non-pancreatic ascites [4]. The combination of black discoloration, high enzymatic content, and lack of evidence of infection or malignancy underscores the exceptional nature of this case.

The management of pancreatic ascites follows a stepwise approach. Conservative treatment includes fluid and electrolyte replacement, enteral nutrition, percutaneous drainage, and monitoring for complications [3,5]. If conservative therapy fails, endoscopic retrograde cholangiopancreatography (ERCP) with sphincterotomy and transpapillary pancreatic duct stenting is the preferred next step, as it promotes drainage into the duodenum and facilitates ductal healing [4,5]. In cases where endoscopic treatment is not feasible or unsuccessful, surgical options such as internal or external drainage procedures may be required [5].

In our case, conservative management with ascitic drainage was attempted and was technically successful. However, the patient's condition continued to deteriorate. He developed multiple organ dysfunction syndrome (MODS), characterized by respiratory failure requiring mechanical ventilation, refractory hypotension despite vasopressor support, and worsening renal function necessitating continuous hemodiafiltration. MODS is a well-known predictor of poor prognosis in severe acute pancreatitis, and it ultimately led to the patient's death in this case. Due to his unstable hemodynamic status, further diagnostic imaging or interventional procedures could not be pursued. Concerns about the possible exacerbation of pancreatitis by ERCP led us to withhold endoscopic intervention. Surgical options were also deemed inappropriate due to the high invasiveness and the need for precise planning in the hyperacute phase. In retrospect, the use of circulatory support devices might have enabled the safe performance of endoscopic pancreatic duct drainage.

## Conclusions

In cases of acute pancreatitis with persistent hemodynamic instability in the hyperacute phase, ascitic paracentesis should be considered. While ascitic fluid in acute pancreatitis is typically pale yellow and exudative, the presence of black pancreatic ascites should also be recognized as a possibility. In such cases, ascitic drainage is the first-line treatment. If clinical improvement remains inadequate, pancreatic duct stenting should be considered as a potential next step.
